# 5-Cyclo­hexyl-2-methyl-3-(4-methyl­phenyl­sulfon­yl)-1-benzofuran

**DOI:** 10.1107/S1600536812010434

**Published:** 2012-03-14

**Authors:** Hong Dae Choi, Pil Ja Seo, Uk Lee

**Affiliations:** a Department of Chemistry, Dongeui University, San 24 Kaya-dong Busanjin-gu, Busan 614-714, Republic of Korea; b Department of Chemistry, Pukyong National University, 599-1 Daeyeon 3-dongNam-gu, Busan 608-737, Republic of Korea

## Abstract

In the title compound, C_22_H_24_O_3_S, the cyclo­hexyl ring adopts a chair conformation. The 4-methyl­phenyl ring makes a dihedral angle of 80.95 (4)° with the mean plane [mean deviation = 0.011 (1) Å] of the benzofuran fragment. In the crystal, mol­ecules are linked by weak C—H⋯O and C—H⋯π inter­actions.

## Related literature

For background information and the crystal structures of the related compounds, see: Choi *et al.* (2011 ▶, 2012 ▶).
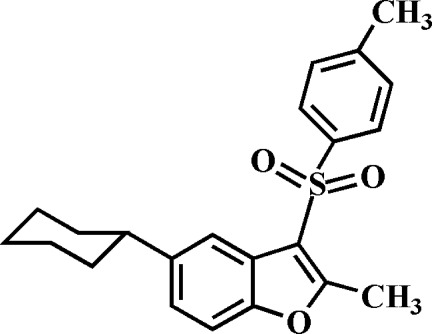


## Experimental

### 

#### Crystal data

C_22_H_24_O_3_S
*M* *_r_* = 368.47
Monoclinic, 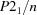

*a* = 10.6806 (2) Å
*b* = 12.5869 (3) Å
*c* = 14.2736 (3) Å
β = 93.094 (1)°
*V* = 1916.08 (7) Å^3^
*Z* = 4
Mo *K*α radiation
μ = 0.19 mm^−1^
*T* = 173 K
0.36 × 0.26 × 0.24 mm

#### Data collection

Bruker SMART APEXII CCD diffractometer
Absorption correction: multi-scan (*SADABS*; Bruker, 2009 ▶) *T*_min_ = 0.936, *T*_max_ = 0.956
19350 measured reflections
4762 independent reflections
3810 reflections with *I* > 2σ(*I*)
*R*_int_ = 0.034

#### Refinement

*R*[*F* ^2^ > 2σ(*F* ^2^)] = 0.042
*wR*(*F* ^2^) = 0.118
*S* = 1.05
4762 reflections
238 parameters
H-atom parameters constrained
Δρ_max_ = 0.29 e Å^−3^
Δρ_min_ = −0.38 e Å^−3^

### 

Data collection: *APEX2* (Bruker, 2009 ▶); cell refinement: *SAINT* (Bruker, 2009 ▶); data reduction: *SAINT*; program(s) used to solve structure: *SHELXS97* (Sheldrick, 2008 ▶); program(s) used to refine structure: *SHELXL97* (Sheldrick, 2008 ▶); molecular graphics: *ORTEP-3* (Farrugia, 1997 ▶) and *DIAMOND* (Brandenburg, 1998 ▶); software used to prepare material for publication: *SHELXL97*.

## Supplementary Material

Crystal structure: contains datablock(s) global, I. DOI: 10.1107/S1600536812010434/fb2242sup1.cif

Structure factors: contains datablock(s) I. DOI: 10.1107/S1600536812010434/fb2242Isup2.hkl

Supplementary material file. DOI: 10.1107/S1600536812010434/fb2242Isup3.cml

Additional supplementary materials:  crystallographic information; 3D view; checkCIF report

## Figures and Tables

**Table 1:** Hydrogen-bond geometry (Å, °) *Cg*1 and *Cg*2 are the centroids of the C2–C7 benzene and the C1/C2/C7/O1/C8 furan rings, respectively.

*D*—H⋯*A*	*D*—H	H⋯*A*	*D*⋯*A*	*D*—H⋯*A*
C6—H6⋯O2^i^	0.95	2.52	3.3888 (19)	152
C13—H13*B*⋯O2^ii^	0.99	2.55	3.447 (2)	151
C10—H10*A*⋯*Cg*1^iii^	0.99	2.73	3.675 (2)	159
C11—H11*A*⋯*Cg*2^iii^	0.99	2.95	3.646 (2)	128
C20—H20⋯*Cg*1^iv^	0.95	2.82	3.745 (2)	164
C22—H22*C*⋯*Cg*2^iv^	0.98	2.96	3.907 (2)	164

Symmetry codes: (i) 
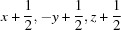
; (ii) 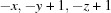; (iii) 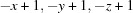; (iv) 
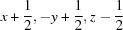
.

**Fig. 1.**
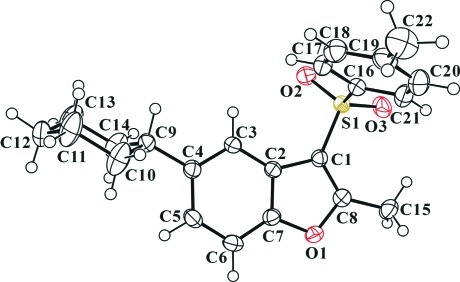
The title molecule with the atom numbering scheme. The displacement ellipsoids are drawn at the 50% probability level. H atoms are presented as small spheres of arbitrary radius.

**Fig. 2.**
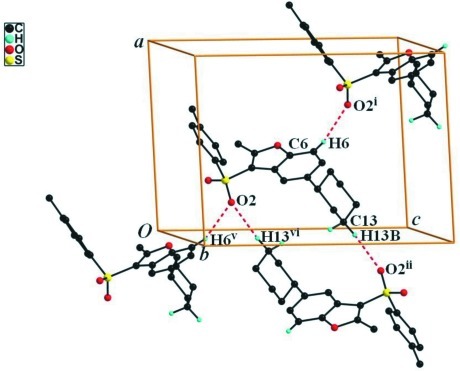
A view of the C—H···O interactions (dotted lines) in the title crystal structure. The H atoms non-participating in the hydrogen-bond pattern were omitted for clarity. [Symmetry codes: (i) x + 1/2, -y + 1/2, z + 1/2; (ii) -x, -y + 1 , -z + 1; (iv) x - 1/2, -y + 1/2, z - 1/2; (v) -x, -y + 1, -z +1.]

**Fig. 3.**
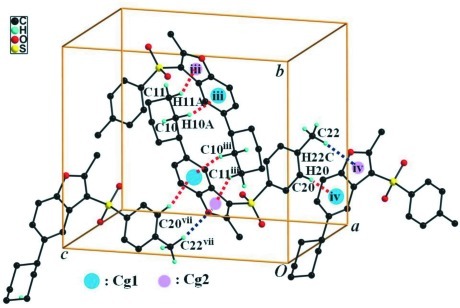
C—H···π-electron ring interactions (dotted lines) in the title crystal structure. The H atoms non-participating in hydrogen-bond pattern were omitted for clarity. [Symmetry codes: (iii) -x + 1, -y + 1, -z + 1; (vi) x + 1/2, -y + 1/2, z - 1/2; (vii) x - 1/2, -y + 1/2, z + 1/2.]

## supplementary crystallographic information

### Comment

As a part of our ongoing study of 5-cyclohexyl-2-methyl-1-benzofuran derivatives containing either 3-(4-fluorophenylsulfonyl) (Choi *et al.*, 2011) or 3-(4-bromophenylsulfonyl) (Choi *et al.*, 2012) substituents, we report herein the crystal structure of the title compound.

In the title molecule (Fig. 1), the benzofuran unit is essentially planar, with a mean deviation of 0.011 (1) Å from the least-squares plane defined by the nine non-hydrogen constituent atoms. The cyclohexyl ring is in the chair conformation. The dihedral angle between the 4-methylphenyl ring and the mean plane of the benzofuran fragment is 80.95 (4)°. The crystal packing is stabilized by weak intermolecular C—H···O hydrogen bonds (Fig. 2; Table 1). The crystal packing is further stabilized by intermolecular C—H···π-electron ring interactions (Fig. 3; Table 1, Cg1 and Cg2 are the centroids of the C2–C7 benzene ring and the C1/C2/C7/O1/C8 furan ring, respectively).

### Experimental

77% 3-chloroperoxybenzoic acid - the product of Aldrich Chemical Co. - (381 mg, 1.7 mmol) was added in small portions to a stirred solution of 5-cyclohexyl-2-methyl-3-(4-methylphenylsulfanyl)-1-benzofuran (269 mg, 0.8 mmol) in dichloromethane (30 ml) at 273 K. After being stirred at room temperature for 8h, the mixture was washed with saturated solution of of NaHCO_3_ and the organic layer was separated, dried over MgSO_4_, filtered and concentrated at reduced pressure. The residue was purified by column chromatography (hexane–ethyl acetate, 4:1 v/v) to afford the title compound as a colorless solid [yield 72%, m.p. 437–438 K; *R*_f_ = 0.48 (hexane–ethyl acetate, 4:1 v/v)]. Single crystals suitable for X-ray diffraction were prepared by slow evaporation of a solution of the title compound in benzene at room temperature. The average crystal size was approximatly 1.2 × 1.0 × 0.7 mm. (The measureed crystal was cut from the large one.) The crystals are colourless and soluble in polar solvents.

### Refinement

All the hydrogens were discerned in the difference electron density maps. However, they were situated into the idealized positions and refined using a riding model, with C—H = 0.95, 1.0, 0.99 and 0.98 Å for aryl, methine, methylene and methyl H atoms, respectively. *U*_iso_(H) =1.2*U*_eq_(C) for aryl, methine, and methylene, and 1.5*U*_eq_(C) for methyl H atoms. The positions of methyl hydrogens were optimized rotationally.

### Crystal data

**Table:**

C_22_H_24_O_3_S	*F*(000) = 784
*M**_r_* = 368.47	*D*_x_ = 1.277 Mg m^−^^3^
Monoclinic, *P*2_1_/*n*	Melting point = 437–438 K
Hall symbol: -P 2yn	Mo *K*α radiation, λ = 0.71073 Å
*a* = 10.6806 (2) Å	Cell parameters from 5853 reflections
*b* = 12.5869 (3) Å	θ = 2.2–27.7°
*c* = 14.2736 (3) Å	µ = 0.19 mm^−^^1^
β = 93.094 (1)°	*T* = 173 K
*V* = 1916.08 (7) Å^3^	Block, colourless
*Z* = 4	0.36 × 0.26 × 0.24 mm

### Data collection

**Table:**

Bruker SMART APEXII CCD diffractometer	4762 independent reflections
Radiation source: rotating anode	3810 reflections with *I* > 2σ(*I*)
Graphite multilayer monochromator	*R*_int_ = 0.034
Detector resolution: 10.0 pixels mm^-1^	θ_max_ = 28.3°, θ_min_ = 2.2°
φ and ω scans	*h* = −14→13
Absorption correction: multi-scan (*SADABS*; Bruker, 2009)	*k* = −16→16
*T*_min_ = 0.936, *T*_max_ = 0.956	*l* = −18→19
19350 measured reflections	

### Refinement

**Table:**

Refinement on *F*^2^	Secondary atom site location: difference Fourier map
Least-squares matrix: full	Hydrogen site location: difference Fourier map
*R*[*F*^2^ > 2σ(*F*^2^)] = 0.042	H-atom parameters constrained
*wR*(*F*^2^) = 0.118	*w* = 1/[σ^2^(*F*_o_^2^) + (0.0573*P*)^2^ + 0.6267*P*] where *P* = (*F*_o_^2^ + 2*F*_c_^2^)/3
*S* = 1.05	(Δ/σ)_max_ < 0.001
4762 reflections	Δρ_max_ = 0.29 e Å^−^^3^
238 parameters	Δρ_min_ = −0.38 e Å^−^^3^
0 restraints	Extinction correction: *SHELXL97* (Sheldrick, 2008), Fc^*^=kFc[1+0.001xFc^2^λ^3^/sin(2θ)]^-1/4^
94 constraints	Extinction coefficient: 0.0052 (10)
Primary atom site location: structure-invariant direct methods	

### Special details

**Table:**

Geometry. All esds (except the esd in the dihedral angle between two l.s. planes) are estimated using the full covariance matrix. The cell esds are taken into account individually in the estimation of esds in distances, angles and torsion angles; correlations between esds in cell parameters are only used when they are defined by crystal symmetry. An approximate (isotropic) treatment of cell esds is used for estimating esds involving l.s. planes.
Refinement. Refinement of F^2^ against ALL reflections. The weighted R-factor wR and goodness of fit S are based on F^2^, conventional R-factors R are based on F, with F set to zero for negative F^2^. The threshold expression of F^2^ > 2sigma(F^2^) is used only for calculating R-factors(gt) etc. and is not relevant to the choice of reflections for refinement. R-factors based on F^2^ are statistically about twice as large as those based on F, and R- factors based on ALL data will be even larger.

### Fractional atomic coordinates and isotropic or equivalent isotropic displacement parameters (Å^2^)

**Table:**

	*x*	*y*	*z*	*U*_iso_*/*U*_eq_	
S1	0.27825 (3)	0.22598 (3)	0.23844 (2)	0.02775 (12)	
O1	0.44984 (10)	0.12891 (8)	0.47290 (7)	0.0312 (2)	
O2	0.16711 (10)	0.28891 (9)	0.24653 (8)	0.0348 (3)	
O3	0.27066 (12)	0.12657 (9)	0.18864 (8)	0.0392 (3)	
C1	0.34066 (13)	0.20282 (11)	0.35128 (10)	0.0257 (3)	
C2	0.33675 (13)	0.27597 (11)	0.42945 (10)	0.0246 (3)	
C3	0.28562 (13)	0.37621 (11)	0.44468 (10)	0.0262 (3)	
H3	0.2377	0.4114	0.3960	0.031*	
C4	0.30579 (14)	0.42369 (12)	0.53198 (10)	0.0286 (3)	
C5	0.37647 (17)	0.36927 (13)	0.60298 (11)	0.0354 (4)	
H5	0.3897	0.4024	0.6625	0.042*	
C6	0.42743 (16)	0.26992 (13)	0.58987 (11)	0.0349 (4)	
H6	0.4746	0.2338	0.6384	0.042*	
C7	0.40552 (14)	0.22645 (11)	0.50199 (10)	0.0278 (3)	
C8	0.40998 (14)	0.11734 (12)	0.38090 (11)	0.0289 (3)	
C9	0.25195 (15)	0.53261 (12)	0.54981 (10)	0.0293 (3)	
H9	0.2209	0.5618	0.4877	0.035*	
C10	0.34893 (17)	0.60968 (14)	0.59082 (18)	0.0542 (5)	
H10A	0.4183	0.6159	0.5479	0.065*	
H10B	0.3841	0.5818	0.6515	0.065*	
C11	0.2925 (2)	0.71952 (15)	0.60601 (19)	0.0599 (6)	
H11A	0.3572	0.7670	0.6354	0.072*	
H11B	0.2642	0.7505	0.5447	0.072*	
C12	0.1826 (2)	0.71215 (16)	0.66823 (14)	0.0532 (5)	
H12A	0.2123	0.6875	0.7314	0.064*	
H12B	0.1448	0.7834	0.6748	0.064*	
C13	0.08508 (19)	0.63650 (15)	0.62771 (16)	0.0521 (5)	
H13A	0.0491	0.6652	0.5676	0.063*	
H13B	0.0164	0.6302	0.6713	0.063*	
C14	0.14023 (17)	0.52678 (14)	0.61102 (15)	0.0443 (4)	
H14A	0.1667	0.4943	0.6721	0.053*	
H14B	0.0750	0.4807	0.5804	0.053*	
C15	0.45123 (17)	0.01826 (13)	0.33566 (13)	0.0396 (4)	
H15A	0.4121	−0.0430	0.3648	0.059*	
H15B	0.5426	0.0121	0.3437	0.059*	
H15C	0.4262	0.0202	0.2686	0.059*	
C16	0.39138 (14)	0.30592 (12)	0.18739 (10)	0.0289 (3)	
C17	0.38998 (15)	0.41549 (13)	0.19887 (11)	0.0335 (3)	
H17	0.3265	0.4485	0.2328	0.040*	
C18	0.48222 (16)	0.47598 (14)	0.16024 (12)	0.0391 (4)	
H18	0.4812	0.5510	0.1678	0.047*	
C19	0.57661 (16)	0.42936 (15)	0.11046 (12)	0.0397 (4)	
C20	0.57578 (17)	0.31987 (16)	0.09985 (13)	0.0451 (4)	
H20	0.6392	0.2869	0.0659	0.054*	
C21	0.48410 (16)	0.25753 (14)	0.13786 (12)	0.0387 (4)	
H21	0.4848	0.1826	0.1301	0.046*	
C22	0.67779 (19)	0.49602 (19)	0.07082 (16)	0.0586 (6)	
H22A	0.7388	0.5166	0.1213	0.088*	
H22B	0.6405	0.5599	0.0418	0.088*	
H22C	0.7201	0.4550	0.0235	0.088*	

### Atomic displacement parameters (Å^2^)

**Table:**

	*U*^11^	*U*^22^	*U*^33^	*U*^12^	*U*^13^	*U*^23^
S1	0.0288 (2)	0.0292 (2)	0.02484 (19)	0.00142 (14)	−0.00237 (13)	−0.00406 (13)
O1	0.0351 (6)	0.0265 (5)	0.0316 (5)	0.0055 (4)	−0.0032 (4)	0.0009 (4)
O2	0.0266 (5)	0.0437 (6)	0.0334 (6)	0.0046 (5)	−0.0036 (4)	−0.0028 (5)
O3	0.0504 (7)	0.0344 (6)	0.0324 (6)	−0.0037 (5)	−0.0025 (5)	−0.0098 (5)
C1	0.0250 (7)	0.0257 (7)	0.0263 (7)	0.0003 (5)	0.0009 (5)	−0.0021 (5)
C2	0.0245 (7)	0.0258 (7)	0.0234 (6)	−0.0020 (5)	0.0006 (5)	0.0002 (5)
C3	0.0279 (7)	0.0279 (7)	0.0228 (6)	0.0018 (5)	0.0013 (5)	0.0018 (5)
C4	0.0331 (8)	0.0264 (7)	0.0264 (7)	0.0002 (6)	0.0042 (6)	0.0008 (6)
C5	0.0490 (10)	0.0339 (8)	0.0228 (7)	0.0011 (7)	−0.0030 (6)	−0.0022 (6)
C6	0.0425 (9)	0.0354 (8)	0.0261 (7)	0.0042 (7)	−0.0058 (6)	0.0038 (6)
C7	0.0292 (7)	0.0253 (7)	0.0288 (7)	0.0016 (5)	0.0005 (6)	0.0024 (6)
C8	0.0274 (7)	0.0283 (7)	0.0310 (7)	−0.0013 (6)	0.0011 (6)	−0.0005 (6)
C9	0.0371 (8)	0.0269 (7)	0.0243 (7)	0.0029 (6)	0.0045 (6)	0.0002 (6)
C10	0.0347 (9)	0.0336 (9)	0.0952 (16)	−0.0068 (7)	0.0131 (10)	−0.0150 (10)
C11	0.0483 (11)	0.0318 (10)	0.1003 (18)	−0.0080 (8)	0.0102 (11)	−0.0207 (10)
C12	0.0756 (14)	0.0419 (10)	0.0426 (10)	0.0152 (10)	0.0083 (10)	−0.0107 (8)
C13	0.0461 (11)	0.0418 (10)	0.0712 (13)	0.0083 (8)	0.0295 (10)	0.0054 (9)
C14	0.0369 (9)	0.0333 (9)	0.0642 (12)	−0.0006 (7)	0.0166 (8)	0.0030 (8)
C15	0.0431 (9)	0.0305 (8)	0.0445 (9)	0.0099 (7)	−0.0034 (7)	−0.0066 (7)
C16	0.0304 (7)	0.0321 (8)	0.0239 (7)	0.0042 (6)	0.0000 (6)	−0.0004 (6)
C17	0.0341 (8)	0.0329 (8)	0.0334 (8)	0.0054 (6)	0.0023 (6)	−0.0033 (6)
C18	0.0410 (9)	0.0347 (9)	0.0416 (9)	−0.0014 (7)	0.0008 (7)	−0.0002 (7)
C19	0.0340 (9)	0.0502 (10)	0.0348 (8)	−0.0013 (7)	0.0007 (7)	0.0041 (7)
C20	0.0390 (9)	0.0526 (11)	0.0449 (10)	0.0085 (8)	0.0132 (8)	−0.0012 (8)
C21	0.0411 (9)	0.0350 (8)	0.0406 (9)	0.0089 (7)	0.0083 (7)	−0.0029 (7)
C22	0.0437 (11)	0.0731 (15)	0.0596 (13)	−0.0112 (10)	0.0084 (9)	0.0082 (11)

### Geometric parameters (Å, º)

**Table:**

S1—O2	1.4368 (11)	C11—H11B	0.9900
S1—O3	1.4391 (11)	C12—C13	1.504 (3)
S1—C1	1.7343 (15)	C12—H12A	0.9900
S1—C16	1.7603 (16)	C12—H12B	0.9900
O1—C8	1.3663 (18)	C13—C14	1.525 (2)
O1—C7	1.3874 (17)	C13—H13A	0.9900
C1—C8	1.360 (2)	C13—H13B	0.9900
C1—C2	1.4490 (19)	C14—H14A	0.9900
C2—C7	1.385 (2)	C14—H14B	0.9900
C2—C3	1.396 (2)	C15—H15A	0.9800
C3—C4	1.388 (2)	C15—H15B	0.9800
C3—H3	0.9500	C15—H15C	0.9800
C4—C5	1.408 (2)	C16—C21	1.388 (2)
C4—C9	1.514 (2)	C16—C17	1.389 (2)
C5—C6	1.381 (2)	C17—C18	1.383 (2)
C5—H5	0.9500	C17—H17	0.9500
C6—C7	1.377 (2)	C18—C19	1.394 (2)
C6—H6	0.9500	C18—H18	0.9500
C8—C15	1.482 (2)	C19—C20	1.386 (3)
C9—C10	1.514 (2)	C19—C22	1.503 (3)
C9—C14	1.518 (2)	C20—C21	1.388 (2)
C9—H9	1.0000	C20—H20	0.9500
C10—C11	1.528 (3)	C21—H21	0.9500
C10—H10A	0.9900	C22—H22A	0.9800
C10—H10B	0.9900	C22—H22B	0.9800
C11—C12	1.512 (3)	C22—H22C	0.9800
C11—H11A	0.9900		
			
O2—S1—O3	119.62 (7)	C13—C12—C11	110.87 (16)
O2—S1—C1	107.21 (7)	C13—C12—H12A	109.5
O3—S1—C1	108.73 (7)	C11—C12—H12A	109.5
O2—S1—C16	107.86 (7)	C13—C12—H12B	109.5
O3—S1—C16	108.31 (7)	C11—C12—H12B	109.5
C1—S1—C16	104.03 (7)	H12A—C12—H12B	108.1
C8—O1—C7	106.75 (11)	C12—C13—C14	111.60 (17)
C8—C1—C2	107.52 (13)	C12—C13—H13A	109.3
C8—C1—S1	126.65 (11)	C14—C13—H13A	109.3
C2—C1—S1	125.65 (11)	C12—C13—H13B	109.3
C7—C2—C3	119.11 (13)	C14—C13—H13B	109.3
C7—C2—C1	104.68 (12)	H13A—C13—H13B	108.0
C3—C2—C1	136.20 (13)	C9—C14—C13	111.61 (14)
C4—C3—C2	119.05 (13)	C9—C14—H14A	109.3
C4—C3—H3	120.5	C13—C14—H14A	109.3
C2—C3—H3	120.5	C9—C14—H14B	109.3
C3—C4—C5	119.19 (14)	C13—C14—H14B	109.3
C3—C4—C9	119.93 (13)	H14A—C14—H14B	108.0
C5—C4—C9	120.89 (13)	C8—C15—H15A	109.5
C6—C5—C4	122.88 (14)	C8—C15—H15B	109.5
C6—C5—H5	118.6	H15A—C15—H15B	109.5
C4—C5—H5	118.6	C8—C15—H15C	109.5
C7—C6—C5	115.76 (14)	H15A—C15—H15C	109.5
C7—C6—H6	122.1	H15B—C15—H15C	109.5
C5—C6—H6	122.1	C21—C16—C17	120.55 (15)
C6—C7—C2	124.01 (14)	C21—C16—S1	118.98 (12)
C6—C7—O1	125.47 (14)	C17—C16—S1	120.45 (11)
C2—C7—O1	110.51 (13)	C18—C17—C16	119.15 (15)
C1—C8—O1	110.52 (13)	C18—C17—H17	120.4
C1—C8—C15	134.40 (14)	C16—C17—H17	120.4
O1—C8—C15	115.08 (13)	C17—C18—C19	121.45 (16)
C4—C9—C10	112.78 (13)	C17—C18—H18	119.3
C4—C9—C14	111.75 (13)	C19—C18—H18	119.3
C10—C9—C14	110.55 (14)	C20—C19—C18	118.27 (16)
C4—C9—H9	107.1	C20—C19—C22	120.96 (17)
C10—C9—H9	107.1	C18—C19—C22	120.76 (18)
C14—C9—H9	107.1	C19—C20—C21	121.32 (16)
C9—C10—C11	111.57 (16)	C19—C20—H20	119.3
C9—C10—H10A	109.3	C21—C20—H20	119.3
C11—C10—H10A	109.3	C20—C21—C16	119.26 (16)
C9—C10—H10B	109.3	C20—C21—H21	120.4
C11—C10—H10B	109.3	C16—C21—H21	120.4
H10A—C10—H10B	108.0	C19—C22—H22A	109.5
C12—C11—C10	110.64 (17)	C19—C22—H22B	109.5
C12—C11—H11A	109.5	H22A—C22—H22B	109.5
C10—C11—H11A	109.5	C19—C22—H22C	109.5
C12—C11—H11B	109.5	H22A—C22—H22C	109.5
C10—C11—H11B	109.5	H22B—C22—H22C	109.5
H11A—C11—H11B	108.1		
			
O2—S1—C1—C8	151.36 (13)	C7—O1—C8—C15	178.36 (13)
O3—S1—C1—C8	20.71 (16)	C3—C4—C9—C10	−128.70 (17)
C16—S1—C1—C8	−94.55 (14)	C5—C4—C9—C10	51.3 (2)
O2—S1—C1—C2	−34.09 (14)	C3—C4—C9—C14	106.03 (17)
O3—S1—C1—C2	−164.74 (12)	C5—C4—C9—C14	−73.9 (2)
C16—S1—C1—C2	80.00 (13)	C4—C9—C10—C11	178.75 (17)
C8—C1—C2—C7	−0.40 (16)	C14—C9—C10—C11	−55.3 (2)
S1—C1—C2—C7	−175.82 (11)	C9—C10—C11—C12	56.7 (3)
C8—C1—C2—C3	178.17 (16)	C10—C11—C12—C13	−56.5 (3)
S1—C1—C2—C3	2.8 (3)	C11—C12—C13—C14	56.0 (2)
C7—C2—C3—C4	0.4 (2)	C4—C9—C14—C13	−179.20 (16)
C1—C2—C3—C4	−178.05 (15)	C10—C9—C14—C13	54.3 (2)
C2—C3—C4—C5	−0.4 (2)	C12—C13—C14—C9	−55.1 (2)
C2—C3—C4—C9	179.60 (13)	O2—S1—C16—C21	−154.15 (13)
C3—C4—C5—C6	0.1 (3)	O3—S1—C16—C21	−23.34 (15)
C9—C4—C5—C6	−179.94 (15)	C1—S1—C16—C21	92.22 (14)
C4—C5—C6—C7	0.3 (3)	O2—S1—C16—C17	27.71 (14)
C5—C6—C7—C2	−0.4 (2)	O3—S1—C16—C17	158.52 (13)
C5—C6—C7—O1	178.60 (15)	C1—S1—C16—C17	−85.92 (14)
C3—C2—C7—C6	0.0 (2)	C21—C16—C17—C18	0.0 (2)
C1—C2—C7—C6	178.91 (15)	S1—C16—C17—C18	178.14 (13)
C3—C2—C7—O1	−179.07 (12)	C16—C17—C18—C19	−0.3 (3)
C1—C2—C7—O1	−0.20 (16)	C17—C18—C19—C20	0.4 (3)
C8—O1—C7—C6	−178.37 (15)	C17—C18—C19—C22	−178.60 (17)
C8—O1—C7—C2	0.72 (16)	C18—C19—C20—C21	−0.3 (3)
C2—C1—C8—O1	0.87 (17)	C22—C19—C20—C21	178.70 (18)
S1—C1—C8—O1	176.23 (10)	C19—C20—C21—C16	0.1 (3)
C2—C1—C8—C15	−178.30 (17)	C17—C16—C21—C20	0.1 (3)
S1—C1—C8—C15	−2.9 (3)	S1—C16—C21—C20	−178.07 (14)
C7—O1—C8—C1	−0.98 (16)		

### Hydrogen-bond geometry (Å, º)

Cg1 and Cg2 are the centroids of the C2–C7 benzene and the C1/C2/C7/O1/C8 furan rings, respectively.

**Table:**

*D*—H···*A*	*D*—H	H···*A*	*D*···*A*	*D*—H···*A*
C6—H6···O2^i^	0.95	2.52	3.3888 (19)	152
C13—H13*B*···O2^ii^	0.99	2.55	3.447 (2)	151
C10—H10*A*···*Cg*1^iii^	0.99	2.73	3.675 (2)	159
C11—H11*A*···*Cg*2^iii^	0.99	2.95	3.646 (2)	128
C20—H20···*Cg*1^iv^	0.95	2.82	3.745 (2)	164
C22—H22*C*···*Cg*2^iv^	0.98	2.96	3.907 (2)	164

Symmetry codes: (i) *x*+1/2, −*y*+1/2, *z*+1/2; (ii) −*x*, −*y*+1, −*z*+1; (iii) −*x*+1, −*y*+1, −*z*+1; (iv) *x*+1/2, −*y*+1/2, *z*−1/2.
